# Advanced Glycation End Products in the Pathogenesis of Psoriasis

**DOI:** 10.3390/ijms18112471

**Published:** 2017-11-20

**Authors:** Anastasia Papagrigoraki, Martina Maurelli, Micol del Giglio, Paolo Gisondi, Giampiero Girolomoni

**Affiliations:** Section of Dermatology, Department of Medicine, University of Verona, 37126 Verona, Italy; papanastassia78@gmail.com (A.P.); maurelli.martina@gmail.com (M.M.); micol.delgiglio@univr.it (M.d.G.); giampiero.girolomoni@univr.it (G.G.)

**Keywords:** advanced glycation end products, psoriasis, inflammation

## Abstract

Advanced glycation end products (AGEs) are extremely oxidant and biologically reactive compounds, which form through oxidation of sugars, lipids and amino acids to create aldehydes that bind covalently to proteins. AGEs formation and accumulation in human tissues is a physiological process during ageing but it is enhanced in case of persistent hyperglycemia, hyperlipidemia and oxidative or carbonyl stress, which are common in patients with moderate to severe psoriasis. Exogenous AGEs may derive from foods, UV irradiation and cigarette smoking. AGEs elicit biological functions by activating membrane receptors expressed on epithelial and inflammatory cell surface. AGEs amplify inflammatory response by favoring the release of cytokines and chemokines, the production of reactive oxygen species and the activation of metalloproteases. AGEs levels are increased in the skin and blood of patients with severe psoriasis independently of associated metabolic disorders. Intensified glycation of proteins in psoriasis skin might have a role in fueling cutaneous inflammation. In addition, AGEs released from psoriatic skin may increase metabolic and cardiovascular risk in patients with severe disease.

## 1. Introduction

Advanced glycation end products (AGEs) are a vast group of highly oxidant and biologically active compounds formed through a series of chemical chain reactions, to create reactive aldehydes that combine covalently to proteins and precipitate in human tissues [1,2]. AGEs formation is a physiological process, with a consequently slow accumulation in human tissues during ageing. However, more rapid and intense accumulation occur in patients with constant hyperglycemia and persistent oxidative or carbonyl stress [1]. AGEs have been implicated in the pathophysiology of inflammatory and metabolic disorders. Here, we review the role of AGEs in psoriasis and in some diseases commonly associated with psoriasis.

## 2. Advanced Glycation End Products (AGEs)

**Figure 1:**
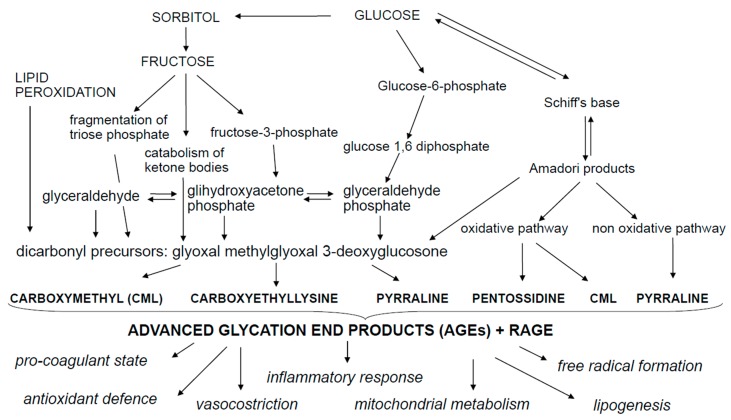
Metabolic pathways leading to the advanced glycation end products (AGEs) production.

AGEs are a group of cross-linked irreversible ketone adducts created by a non-enzymatic glycation between free amino groups of proteins, lipids or nucleic acids and reducing sugars (Figure 1). These interactions form an unstable aldimine compound, the Schiff base; this reaction may occur from 3 to 24 h and is a reversible process [2]. The Schiff base can be rearranged, producing the Amadori compound, which is a non-reversible and stable product and accumulates on proteins over a period of a few days or weeks. The Amadori product undergoes oxidative degradation to generate highly reactive intermediate dicarbonyl compounds, the so called glyoxal, methyglyoxal, deoxyglucosones, that interact again with free amino groups of proteins, amplifying the Maillard reaction. According to the classical view of the Maillard reaction, glucose reacts with a primary amine, which is then followed by a series of rearrangements and/or fragmentation reactions to yield the final AGEs. The main modified residues are *N*-carboxymethylisine and *N*-carboxylethylisine (CML and CEL), *N*-lactatolisine, pyrraline, pentosidine and imidazoles [3,4]. The most representative and studied AGEs are CML, CEL and pentosidine. An important mediator involved in the formation of CML and CEL is methylglyoxal (MG). MG is an enzymatic reagent that functions as a glucose-responsive metabolite. It is formed through a non-oxidative process during anaerobic glycolysis via oxidative decomposition of fructose and polyunsaturated fatty acids through the fragmentation of triose phosphate, the catabolism of threonine and the ketolysis [5]. The most frequently observed AGE is pentosidine—formed during the Maillard reaction—following the reaction between ribose sugar and lysin and arginine amino acids presents in collagen [5,6,7,8,9]. CML is created via reaction of side chains of lysine with glyoxal and of oxidative hydrolysis of fructosamines and its peculiarity is that may be formed by additional pathways which include the autoxidation of aldoses and ketoses and the precursors of ascorbic acid, polyunsaturated fatty acids and the dicarbonyl pathways [4,10]. CEL is structurally similar to CML and is formed through a side chain reaction between lysine and methylglyoxal, triosophosphates or produced in age-related lipid peroxidation. Both CML and CEL are biomarkers of oxidative stress resulting from sugar and lipid peroxidation [4]. When glycation is accompanied with oxidation, the products that formed take the name of AGEs. Additionally, advanced lipoxidation end-products (ALEs) can be formed by a non-enzymatic reaction between reactive carbonyl species, generated by lipid peroxidation and lipid metabolism, with the nucleophilic residues of macromolecules [10,11,12]. ALEs may exert detrimental bioactivity through covalent modification of their target proteins and enzymes, which may result in loss of their biologic functions [12,13].

AGEs formation is a physiological process that is part of normal metabolism during lifetime and accumulates slowly in human tissues during ageing. However, more rapid and intense accumulation occurs in association with consistent hyperglycemia and enhanced oxidative or carbonyl stress. Other than endogenous AGEs, exogenous sources may derive from foods (Dietary AGEs-dAGEs), ultraviolet (UV) irradiation and smoking habit. dAGEs are formed from Maillard’s reaction and may be amplified from 10 to 100 times during cooking procedures involving high dry heat temperatures such as grilled and fried food [1,5]. The oxidative stress deriving from UV skin exposure and smoking habit promotes AGEs accumulation by a greater production of oxygen free radicals associated to these toxic environmental agents [1,14,15].

AGEs exert their biological functions by activating membrane receptors (RAGE). RAGE belong to the superfamily of the immunoglobulin and are expressed on the surface of many cell types including epithelial cells (keratinocytes, hepatocytes) and dendritic cells, endothelial cells, monocytes, macrophages, smooth muscle cells, podocytes and astrocyte [4,5,16]. RAGE is also present in both soluble form and bound to the extracellular matrix [16]. In vitro studies have shown that the AGE-RAGE bond leads to the activation of the transcription factor nuclear factor kappa-light-chain-enhancer of activated B cells (NF-κB), which modulates the transcription of several inflammatory genes [5,16,17]. RAGE can bind also other ligands, such as the high-mobility group box 1 (HMGB1), amyloid beta peptide, fibronectin fragments, degraded extracellular matrix fragments and to the superfamily of S100 proteins, including S100A7 (psoriasin), S100A12 (EN-RAGE) and S100A15 (Koebnerisin) [17,18,19]. It has been shown that HMGB1 levels are increased in the serum of patients affected by severe psoriasis [18]. HMGB1 may favor the shift of T regulatory cells into Th17 cells [20], which are crucial players of psoriasis induction. The interaction between AGE-RAGE, or RAGE-other ligands, on inflammatory cells stabilizes the receptor in the active state and amplifies inflammation by favoring the release of cytokines and chemokines, the production of reactive oxygen species and the activation of metalloproteases. Inflammation in turn is associated with induction of more AGEs, thus spreading the inflammatory response through this vicious circle formation [21].

Excessive AGEs accumulation in tissues has been documented in different disorders particularly in metabolic diseases such as diabetes and obesity and AGEs may have a relevant role in the tissue changes and clinical complications associated with these diseases [8,21,22,23,24,25,26,27,28,29,30,31,32,33,34,35,36].

## 3. AGE, Diabetes Mellitus and Chronic Disorders

Increased serum levels of AGEs have been associated mainly with diabetes, metabolic syndrome and cardiovascular diseases [30,31,32,33] but a role for AGEs has been also hypothesized in Alzheimer’s disease, pancreatic cancer and colorectal cancer [37,38]. Diabetes mellitus in the prototype disease characterized by an exceeded formation of AGEs and AGEs accumulation is involved in cardiovascular morbidity and mortality in diabetic patients. Recently, some authors driven by the known comorbidities of psoriasis, including diabetes, studied the potential association between psoriasis and AGEs [1,4,39].

AGEs are implicated in the development of cardiovascular disease in patients with chronic inflammatory diseases. AGEs increase vascular permeability, neoangiogenesis and stiffness of the arteries and inhibit vasodilation by interfering with nitric oxide, resulting in accelerated atherosclerosis [40,41,42,43,44,45,46,47,48,49,50,51,52,53,54]. AGEs also modify LDLs and promote their uptake by macrophages with foam cell formation [51,52]. The main pathway linking AGEs to cardiovascular risk includes activation of the AGE-RAGE axis with release of oxygen species that induce endothelial cell activation, arterial inflammation, endothelial dysfunction, arterial stiffness, direct myocardial damage and chronic coronary and myocardial inflammation [5,21,40]. Serum levels of AGEs increased and correlated with the severity of cardiovascular disease in patients with type II diabetes mellitus and coronary heart disease [43]. An increased expression of RAGE is associated with an increase in inflammatory reactions in the atherosclerotic carotid plaque and it has been shown that the treatment with statins decreases inflammation and expression of RAGE [45,46]. AGEs accumulation in tissues correlates with prognosis. AGEs levels on skin collagen correlate with long-term diabetic complications and early manifestations of nephropathy. In type I diabetes, the amount of skin AGEs, particularly CML, is a strong predictor of the development and progression of microvascular complications including renal disease [41]. Also in patients with type II diabetes mellitus, skin accumulation of AGEs is a strong predictor of diabetic complications independently of HbA1c [32]. Serum levels of AGEs are predictors of heart failure and development of new cardiovascular events [49]. Finally, elevated levels of AGEs correlate with peripheral and autonomic neuropathy with neuropathic feet ulcers and may aggravate endothelial ulceration and dysfunction in patients with diabetic neuropathy [42].

## 4. AGEs and Psoriasis

Psoriasis is due to an abnormal skin directed T-cell responses, which are dominated by Th17-cells. Cytokines (interleukin 17 (IL-17), IL-22, IL-21, tumor necrosis factor α (TNF-α)) released by activated T-cells induce an abnormal production of autocrine growth factors by keratinocytes, which in turn are stimulated to a higher proliferation and turnover rate. This increased proliferation results in greater demand and higher degradation of glucose by the same keratinocytes. It has been suggested that AGEs may play a role in the pathogenesis of psoriasis [1,39]. The skin is one of the most sensitive tissues to changes induced by AGEs and AGEs accumulation in the skin causes increased production of free radicals. This results in increased production of oxidized LDL and peroxidation products in the skin [4,42,55]. In psoriasis, free radical damage may result from their accumulation in the skin and the lack of an effective protection mechanism against oxidative damage. AGEs may favor chronic inflammation with the activation of monocytes, macrophages, neutrophils and endothelial cells; these cells, once activated, produce pro-inflammatory cytokines and other reactive oxygen forms, resulting in molecular modifications and additional production of AGE, with further amplification of the inflammatory response [1,4]. The increased production of free radicals in psoriasis has also been demonstrated in dermal fibroblasts, which exhibit an increased level of carbonyl residues, as evidence of oxidative damage [56]. The oxidative damage and the increased level of MG have a negative effect on the course of psoriasis. A study demonstrated significant correlation between serum levels of MG and psoriasis severity [39]. In addition, oxidative damage is associated with the activation of cytotoxic cytochrome c, which results in induction of keratinocyte apoptosis. Dysregulated keratinocyte apoptosis is relevant to psoriasis pathogenesis [57]. A study conducted by measuring serum AGEs and antibodies against CML and CEL showed significantly increased levels in patients with psoriasis as compared to controls [4]. CML and CEL are biomarkers of oxidative damage resulting from the peroxidation of sugars and lipids. After the remission of the disease, the same patients showed a decrease in serum AGEs. A more recent study showed that cutaneous and serum levels of AGEs in patients with severe psoriasis were significantly higher as compared to patients with mild psoriasis, patients with severe eczema and to healthy controls [1]. In Table S1 are reported the results of the cutaneous AGEs in the various groups of patients. In the same study skin AGEs levels correlated positively with serum AGEs in all patients (*r* = 0.93) and serum AGEs levels well correlated with disease severity in psoriatic patients (*r* = 0.91). On the contrary, serum levels of RAGE were significantly lower in patients with psoriasis compared with those with eczema or healthy individuals and RAGE levels correlated inversely with disease severity evaluated with the PASI score (*r* = −0.71). RAGE appears to be involved in the development of psoriatic plaques and induces pro-inflammatory cytokine secretion and migration of T lymphocytes into the inflammation site [17,58]. The inflammatory response in psoriasis is favored by RAGE binding to multiple ligands, which regulate gene expression by interaction with transcription factors such as NF-κB and activator protein 1 (AP-1) [19]. Tyrosine kinase protein (PKT) is involved in signal transmission from RAGE to NF-κB and contributes to activate keratinocytes to release epithelial and endothelial growth factors. Thus, PTK is a protein which may have an important role in the development of psoriasis [17]. Proteins modified by glycation or glycosidation can also become immunogenic and so amplify pathogenic immune responses [4]. Further ligands of RAGE are represented by the family of S100 proteins, in particular the A7 psoriasis protein (S100A7). The S100A7 psoriasis protein has been discovered and identified in psoriatic plaques and is overexpressed in the skin of patients with psoriasis [59]. In pathological conditions, it is released by keratinocytes and possesses cytokine and antimicrobial activities including the ability to stimulate chemotaxis of granulocytes, monocytes and lymphocytes following binding with its RAGE receptor. In patients with psoriasis, the psoriasin is over-expressed by keratinocytes and binds to RAGE with further AGEs and pentosidine production. The total soluble RAGE levels, which comprise both the extracellular domain of full-length RAGE and the endogenous secreted isoform lacking transmembrane domain (esRAGE), are lower in the serum of patients with psoriasis [1]. The soluble RAGE isoform may in fact counteract the detrimental effects of the full-length isoform by acting as a decoy receptor for its ligands during inflammation. The reduced total RAGE levels in the serum of patients with psoriasis might also be secondary to occupation of RAGE by psoriasin, en-RAGE and Koebnerisin, which are additional S100 proteins up-regulated in the whole epidermis of patients with psoriasis [59,60].

## 5. Conclusions

AGEs formation and accumulation in human tissues causes alterations in cellular structure and function, especially in patients with diabetes, obesity, metabolic syndrome, fatty liver disease and psoriasis. There is strong correlation between AGEs and cardiovascular complications in these diseases. In patients with psoriasis, intensified protein glycation in the skin might have a role in the amplification of skin inflammation and may provide a link between cutaneous inflammation and increased metabolic and cardiovascular risk. Whether AGEs may serve as new therapeutic target for psoriatic patients could be investigated. A series of small molecules have been recently identified that inhibit competitively the cytoplasmatic tail of RAGE. These compounds inhibit in vitro and in vivo RAGE-dependent molecular processes, such as inhibition of RAGE signal transduction, cellular migration, inflammatory gene expression and ischemia-induced perturbation of heart function and may be attractive for the treatment of psoriasis [61].

## Supplementary Materials

The following are available online at www.mdpi.com/1422-0067/18/11/2471/s1.

## Abbreviations

AGEs	Advanced Glycation End Products
CML	Carboxymethyl
CEL	Carboxylethylisine
MG	Methylglyoxal
ALEs	Advanced Lipoxidation End-Products
RAGE	Membrane Receptors of AGEs
PKT	Tyrosine Kinase Protein
