# Supplementary material for: Advanced Glycation End Products in the Pathogenesis of Psoriasis
Source: Int J Mol Sci. 2017 Nov 20;18(11):2471. doi: 10.3390/ijms18112471 (PMC5713437; doi:10.3390/ijms18112471)
Supplement: Supplementary file 1 [file ijms-18-02471-s001.pdf]

Table S1. Skin AGEs levels are increased in patients with severe psoriasis as compared to patients with mild psoriasis, severe eczema and healthy controls [1].

Skin AGEs (a.u.)	Mean	95% CI	SE	SD	*P* Value
Severe Psoriasis	2.87	2.64 – 3.11	0.115	0.619	-
Mild Psoriasis	2.15	1.99 – 2.30	0.075	0.467	0.04
Severe Eczema	1.90	1.73 – 2.07	0.084	0.499	0.02
Healthy Individuals	1.86	1.68 – 2.05	0.09	0.527	0.01

Values are mean ± standard deviation (SD) or standard error (SE) or confidential intervals (CI). Skin AGEs are expressed in arbitrary units (a.u.) and evaluated by the AGE Reader mu® (Diagnoptics Technologies B.V., Groningen, the Netherlands), which measures tissue AGEs by means of fluorescence technique.
